# Adherence to the Mediterranean Diet and Serum Adiponectin Levels in Pregnancy: Results from a Cohort Study in Normal Weight Caucasian Women

**DOI:** 10.3390/nu10070928

**Published:** 2018-07-20

**Authors:** Angela Spadafranca, Gabriele Piuri, Camilla Bulfoni, Isabel Liguori, Alberto Battezzati, Simona Bertoli, Attilio F. Speciani, Enrico Ferrazzi

**Affiliations:** 1 International Center for the Assessment of Nutritional Status, University of Milan, Via Botticelli 21, 20133 Milano, Italy; alberto.battezzati@unimi.it (A.B.); simona.bertoli@unimi.it (S.B.); 2 Department of Clinical and Community Health Sciences, University of Milan, Fondazione IRCCS Ca’ Granda Ospedale Maggiore Policlinico, 20122 Milan, Italy; enrico.ferrazzi@unimi.it; 3 Inflammation Society, 18 Woodlands Park, Bexley DA52EL, UK; gabriele.piuri@me.com (G.P.); attilio.speciani@me.com (A.F.S.); 4 Department of Woman, Mother, and Neonate, Unit of Obstetrics, Buzzi Children’s Hospital, School of Medicine, University of Milan, 20154 Milan, Italy; milla.bulfoni@gmail.com (C.B.); isabel.liguori@gmail.com (I.L.)

**Keywords:** Mediterranean Diet, pregnancy, low-grade inflammation, adiponectin

## Abstract

The Mediterranean Diet (MedDiet) is significantly associated with anti-inflammatory effects and a favorable health outcome. During pregnancy, both inflammatory changes and oxidative balance are essential for a successful outcome, while an unbalanced inflammatory response can be a key mediator of obstetrical syndromes. The aim of this study is to investigate the adherence to MedDiet during pregnancy in the 1st and in the 3rd trimester, and to test whether the adherence was associated with serum adiponectin levels. The study was carried out on 99 normal weight Caucasian women. The adherence to MedDiet was measured by a 13-point Mediterranean scale. The whole sample scored 7.2 ± 1.5, with no difference between first and third trimester (*p* = 0.7). Critical points were: fruit < 3 servings/day in 77% of the sample, beans < 3 times/week in 89%, fish < 2 times/week in 69%, and nut weekly intake < 30 g in 75%. The serum adiponectin levels significantly decreased from the first to the third trimester (−16% ± 4%, *p* = 0.008), which confirms a low-grade inflammatory condition associated with advancing gestational age. The women who were in the highest tertile of the adherence to MedDiet had a lower percentage decrease, as compared with those in the lowest tertile (10% ± 11% vs. −34% ± 3%, *p* = 0.01). Even if in pregnancy the adiponectin levels are strongly influenced by the low-grade inflammation, the adherence to MedDiet may modulate this state.

## 1. Introduction

The Mediterranean Diet (MedDiet), which represents the common dietary pattern among the populations bordering the Mediterranean Sea, has been widely reported to be a model of healthy eating, as it contributes to a favorable health status and a better quality of life [1]. This dietary pattern can be described as a diet characterized by high consumption of plant-based foods, such as fruit, vegetables, whole cereals, nuts, and legumes; low consumption of red-processed meats and dairy products; and moderate consumption of fish and poultry [2], with olive oil as the main source of fat. Polyphenols, vitamins, and fibers derived from plant-based foods, and omega 3 fatty acids derived from fish and nuts, are the main bioactive compounds associated with the anti-inflammatory and antioxidant properties of MedDiet [3]. Although many studies, both in vitro and in vivo, investigated the role of these compounds on inflammatory and oxidative status, over the past few years, the research carried out in this field has been mainly focused on estimating adherence to the whole MedDiet, rather than analyzing the individual components of the dietary pattern [4]. This is because the analyses of single nutrients ignore some important interactions between the different components of a diet and, more importantly, because people do not eat isolated nutrients [1]. Therefore, dietary scores estimating adherence to MedDiet have been found associated with beneficial effects on human health and, in particular, with a reduction of mortality from cardiovascular diseases and cancer [5]. Pregnancy is a delicate period in a woman’s life and is characterized by a low-grade inflammatory condition. The evidence in favor of this is that normal gestation is accompanied by an increased plasma concentration of acute phase reactants (i.e., fibrinogen, plasminogen activator inhibitor-1, ceruloplasmin, IL-12) and an increased number of leukocytes. However, if on the one hand, inflammatory changes and oxidative balance are essential for successful outcome, on the other hand, an unbalanced and exaggerated inflammatory response can be a key mediator of obstetrical syndromes (preeclampsia and preterm labor) [6]. Some studies, carried out in adults, suggest that low-grade inflammation can be mitigated by health-promoting behaviors, such as the healthy eating patterns of MedDiet [7], but no study, to our knowledge, has investigated this topic during pregnancy. 

Adiponectin, an adipose tissue-secreted cytokine, has been shown to improve insulin sensitivity, to regulate glucose and lipid metabolism, and to have pronounced antiatherosclerosis effects in the general population [8,9,10,11]. Pregnancy is a unique situation, in which there is a physiological but temporary increase in insulin resistance [12]; the mechanisms responsible for gestational-induced insulin resistance are not completely understood. Nevertheless, recent studies have reported that, in addition to estrogen, progesterone, cortisol, and human lactogen [13], adipocytokines—as adiponectin, which is produced abundantly and exclusively in the adipose tissue—are possible regulators of insulin resistance and glucose homeostasis in pregnancy. Although there are several studies about the association between adiponectin and gestational diabetes or preeclampsia [14,15,16,17,18], only a few of them have focused their attention on the study of normal pregnant subjects, measuring adiponectin concentration in different trimesters [19,20]. Moreover, some studies have assessed the relationship between adiponectin and adherence to MedDiet in adults and adolescents, highlighting a direct association [21,22], but no study has investigated this relationship in pregnancy.

Hence, the primary objective of the present study is to examine adherence to MedDiet during pregnancy in the first and in the third trimester. A secondary objective is to test whether the adherence to this dietary pattern is associated with serum adiponectin levels. Finally, we tested whether women with high adherence to MedDiet have a less substantial decrease in the adiponectin levels. 

## 2. Materials and Methods

### 2.1. Study Design and Subjects

This study was carried out in a cohort of normal weight Caucasian pregnant women (pre-pregnant body mass index: 18.5–24.9 kg/m^2^) recruited at the Department of Woman, Mother and Neonate, Buzzi Children’s Hospital (Milan, Italy) during the obstetrical and gynecological visits that are planned in the beginning of pregnancy (within 16th gestational week). 

Exclusion criteria were chronic gastrointestinal diseases; pre-pregnant diabetes; celiac disease; history of eating disorders, such as anorexia or bulimia; vegan, vegetarian, or macrobiotic regimens; diet-therapy in progress; and non-Caucasian ethnicity. 

Anthropometric measures, administration of a modified validated food frequency questionnaire to assess adherence to MedDiet, and fasting blood sample collection to measure serum adiponectin were taken 2 days after the visit of recruitment (first trimester) and another time at the visit of the third trimester. 

Study procedures were approved by the institutional review board, and each subject provided written informed consent. The study was carried out according to the Declaration of Helsinki.

### 2.2. Anthropometry

Anthropometric measurements were taken by the same operator at the first trimester and third trimester, according to standard criteria and measuring procedures [23]. Pregnant women were wearing only underwear. Their weight (to the nearest 0.1 kg) and standing height (SH; to the nearest 0.1 cm) were measured using the same calibrated scale, which had a telescopic vertical steel stadiometer (SECA 711, Hamburg, Germany). 

BMI (body mass index) was calculated as weight (kg)/stature (m^2^). Pre-pregnancy BMI was calculated considering self-reported body weight prior to pregnancy. 

### 2.3. Mediterranean Dietary Pattern

Adherence to MedDiet was assessed using a 14-item questionnaire validated in a high-risk adult population [24]. In accordance with the Guidelines for Pregnancy (Istituto Superiore Sanità, Ministero della Salute), which recommend abstinence from alcohol, we did not include alcohol consumption in the index. Thus, we calculated MedScore by taking into account 13 items instead of 14, which was done in previous studies [25,26]. Only the participants who scored 8 points (MedScore), instead of 9, were considered as having a good adherence to MedDiet patterns. Adherence to the Mediterranean Diet is defined according to the following recommendations: olive oil as main culinary lipid, olive oil ≥ 4 tablespoons/day, vegetables ≥ 2 servings/day, fruit ≥ 3 servings/day, legumes ≥ 3 servings/week, fish/seafood ≥ 2 servings/week, tree nuts ≥ 30 g/week, poultry more than red meats, use of sofrito sauce ≥ 2 servings/week, red/processed meat < 1 serving/day, butter, cream, margarine < 1 serving/day, soda drinks < 1 serving/day, and commercial sweets and confectionery < 3 servings/week. In accordance with the recommendations, intake was worth 1 point, while noncompliance with recommendations was worth 0 [24]. The questionnaire was considered valid only when all the items were duly answered. 

### 2.4. Serum Adiponectin Determination 

Participants had their fasting morning blood samples taken on visits during the first trimester (2 days after recruitment) and the third trimester. Serum was first separated and then centrifuged and stored at −80 °C for later analysis. Blood samples were analyzed for serum adiponectin using Quantikine-Human Adiponectin/Acrp30 Immunoassay (R&D Systems Inc., Minneapolis, MN, USA). 

### 2.5. Statistical Analysis

Statistical analysis was performed using SPSS version 23 (IBM SPSS Statistics, IBM Corp., Armonk, NY, USA). All data are expressed as mean ± standard deviation (SD) or error standard (ES) for continuous variables and as % for categorical variables. Continuous variables between the first trimester and third trimester were compared using paired Student’s *t* test, while nominal variables by were assessed by chi-square test. Linear regression analysis, adjusted for BMI, age, and gestational age, was used to investigate the association between MedScore (independent) and adiponectin serum levels, and the association between MedScore and percentage decrease in adiponectin levels from the first to the third trimester. Women were categorized in tertiles of adherence to MedDiet (expressed as mean MedScore between the first and the third trimester). Percentage decreases in adiponectin levels were compared between the extreme of MedScore tertiles using an unpaired *t* test. Significant values were considered for *p* ≤ 0.05. 

## 3. Results

The study was proposed to 127 women, but only 99 completed the study. Seventeen women were excluded for incomplete food frequency questionnaires, and 11 were excluded because they missed one of the two study visits. 

Table 1 shows the general characteristics of the cohort that we examined in the study (*n* = 99).

Table 2 shows the adherence to MedDiet according to the questionnaire results. It can be observed that the mean Med-Score both at the first (14.3 ± 1.6 weeks) and third trimester (37 ± 1.2 weeks) is less than 8, which is the minimum value needed to define good adherence to MedDiet.

No difference was observed between the first and third trimester. Following are the critical points of adherence: low consumption of fruits, fish, legumes, and nuts. Good adherence was observed as far as the use of olive oil as the main lipid is concerned, though the daily intake was lower than recommended. Based on the questionnaire results, only 7% of the sample regularly consumed whole cereal.

Table 3 shows serum adiponectin levels. It can be observed that there was a significant decrease in adiponectin from the first to the third trimester. The percentage decrease in adiponectin levels from the first to the third trimester was 16% ± 4%. 

No significant association between adherence to MedDiet and adiponectin levels was found at either the first trimester nor at the third trimester (first trimester: *β* = 0.1, *p* = 0.3; third trimester: *β* = 0.1, *p* = 0.4), however, we found a significant association between the adherence to MedDiet—expressed as mean MedScore between the first and the third trimester—and the percentage decrease in adiponectin levels (*β* = 0.6, *p* = 0.008). Table 4 shows that if we classify the cohort according to tertiles of adherence to MedDiet, the women with higher adherence showed less of a decrease in adiponectin levels. 

## 4. Discussion

In a cohort of normal weight Caucasian pregnant women, we found that, overall, there was a low adherence to MedDiet and that this adherence did not change throughout pregnancy. Furthermore, we found that adiponectin decreased from the 1st to the 3rd trimester and that there was less of a decrease in adiponectin levels in women with higher adherence to MedDiet. We have decided to investigate the adherence to MedDiet because, even though the literature supports MedDiet having anti-inflammatory effects on human health, there are very few studies that investigate adherence to the diet in pregnancy, where there exists the physiological condition of a low-grade inflammation. Although the participants in our study were normal weight women, with high education levels, living in a country in the Mediterranean area, we did not observe good adherence. In particular, we found poor habits related to consuming the recommended intake of fish, nuts, legumes, whole cereals, and fruits. These are the MedDiet foods associated with lower inflammation [27] because of their bioactive compound content, i.e., omega-3, fiber, vitamins, and polyphenols. Although there are no available data about the adherence to MedDiet in pregnant women in Italy, a recent study showed that the MedDiet consumption patterns in an Italian population aged 15–64, particularly those in the young people, are low. As a consequence, it is believed that there is a need to promote greater awareness of Mediterranean eating habits [28]. The pattern of low consumption of MedDiet foods was observed in our cohort at the first trimester and remained stable until the third trimester. This means that food items included in the calculation of MedScore did not change throughout pregnancy, but we do not know exactly what happened with respect to all dietary habits. Moreover, we believe that not knowing the women’s adherence to MedDiet and dietary habits before pregnancy is an important limitation; symptoms linked to pregnancy evolution, such as nausea or food cravings, could have had an impact on previous eating habits, which we cannot exclude as having affected the inflammatory status during pregnancy. Many observational studies have examined the association between MedDiet and inflammatory markers in healthy persons and, in general, those studies report inverse correlations [29,30,31]. In a subsample of the Nurse’health Study, a Mediterranean diet index score was inversely associated with circulating CRP and IL-6, as well as markers of endothelial dysfunction (the adhesion molecules sICAM-1, sVCAM-1, and soluble E-selectin). Similar findings were reported in the Attica study, which involved 1514 men and 1528 women: the subjects that had the greatest adherence to MedDiet (those in the highest tertile) had 17% lower IL-6 and 20% lower CRP concentrations compared to those in the lowest tertile [31]. Mantzoros et al. [21] reported that adherence to the Mediterranean dietary pattern is positively associated with plasma adiponectin concentrations in diabetic women. As shown, different biomarkers have been investigated in the literature to describe inflammatory status associated to MedDiet pattern, but no study has been conducted in pregnant women. In our study, we measured serum adiponectin because it is involved in the development of pregnancy complications, such as insulin resistance, gestational diabetes mellitus, and preeclampsia. Thus, we investigated whether the adherence to MedDiet could have a link with serum adiponectin levels during pregnancy.

In agreement with previous studies in normal pregnancies [19], we observed that adiponectin decreased from the first trimester to the third, which confirms a low-grade inflammatory condition associated with advancing gestational age; the serum concentrations detected at first and third trimester are similar to those found in other studies [19,20]. Different from Mantzoros et al. [21], we did not find any correlation between adiponectin levels and adherence to MedDiet examined at the first and at third trimester. However, we have to consider that our study was conducted during pregnancy, which means that the reported habits could be referred to this period only, which we believe is too short a time to influence inflammatory markers. Moreover, the low-inflammatory state observed in pregnancy may hide the association between diet and adiponectin levels. It would be useful to plan a prospective longitudinal study, where the relationship between inflammatory markers and adherence to MedDiet is investigated before and during pregnancy. Recently, Sureda et al. [22] discovered that the low adherence to the Mediterranean Diet was directly associated with a worse profile of plasmatic inflammatory markers. In nonpregnant women aged 18–65 years, they found that this association was true for hs-CRP and PAI-1, but it was not valid for adiponectin. Even if no association was found between the adherence to MedDiet and adiponectin levels, it is very interesting to observe that women with higher adherence to MedDiet showed a lower percentage decrease in adiponectin levels from the first to the third trimester. This result suggests that, although in pregnancy the adiponectin levels are probably strongly influenced by the low-grade inflammation, the adherence to MedDiet may modulate this state. Additional studies are needed to better understand the role of adiponectin in the low-grade inflammation observed during pregnancy and its association with dietary patterns.

## 5. Strengths and Limitations

This study provides further information on adherence to MedDiet and inflammatory status during pregnancy. Nevertheless, this study has several limitations. First, there is a potential lack of generalizability of the results due to the sample size and to the great predominance of high education levels in the cohort that we tested. We believe that higher education, coupled with voluntary participation in the study, had an influence on the women’s motivation to take part in the research. Secondly, the present cross-sectional design gives limited ability to elucidate the causal relationship between inflammatory markers and MedDiet patterns: it could be useful to conduct a prospective study about the adherence to the Mediterranean Diet before pregnancy. Thirdly, although adiponectin is influenced by the low-grade inflammation state observed in pregnancy, it is an important marker that it is associated with pregnancy complications and whose decrease in advancing gestational age may be influenced by the diet, as shown in this study. It could be useful to add other inflammatory markers, such as CRP, IL-6, and TNF, to have more information about the relationship between MedDiet patterns and inflammatory response in pregnancy.

## 6. Conclusions

We found that, overall, there was a low adherence to MedDiet in a cohort of normal weight Caucasian pregnant women. The lack of association of adiponectin levels at the first and at third trimester with the adherence to MedDiet suggests that adiponectin levels in pregnancy are likely strongly affected by the inflammation, however, the smaller decrease in adiponectin from the first to the third trimester observed in women with higher adherence to MedDiet suggests a role of this dietary pattern in the modulation of the inflammatory status in advancing gestational age. Our results and recent evidence about the epigenetic action of diet on fetal programming [32] should encourage food education before and during pregnancy. 

## Figures and Tables

**Table 1:** General characteristics of the cohort examined in the study (*n* = 99).

		Mean	SD	Range
Age (years)		33.5	3.7	25–43
Pre-pregnant BMI (kg/m^2^)		21.3	1.7	18.6−24.8
Gestational age at 1st trimester (weeks)		14.3	1.6	11–16
Gestational age at 3rd trimester (weeks)		37	1.2	35–38
Weight gain at 3rd trimester (kg)		12.9	0.4	11–14
		*n*	%	
Nulliparous (*n*, %)		69	70	
Education level (*n*, %)	Secondary school	4	4.1	
	High school	30	30.3	
	Degree	65	65.6	

SD: standard deviation.

**Table 2:** Adherence to the Mediterranean Diet at the first and third trimester (*n* = 99).

	1st Trimester	3rd Trimester	*p*
Mediterranean Score (mean ± SD)	7.2 ± 1.5	7.3 ± 1.4	0.7
Adherence Criterion	%	%	
Use of olive as main culinary lipid	100	100	1
Olive oil ≥ 4 table spoons/day	9	8	0.8
Vegetables ≥ 2 servings/day	82	83	0.9
Fruits ≥ 3 servings/day	22.4	23	0.8
Red/processed meats < 1/day	95	93	0.8
Butter, cream, margarine < 1/day	98	98	1
Soda drinks < 1/day	94	92	0.9
Legumes ≥ 3/week	10	12	0.8
Fish/seafoods ≥ 2/week	30	32	0.8
Commercial sweets and confectionery < 3/week	55	57	0.8
Free nuts (30 g) ≥ 1/week	24	26	0.9
Poultry more than red meats	64	62	0.8
Use of sofrito sauce ≥ 2/week	58	57	0.9

**Table 3:** Serum adiponectin levels at the first trimester and third trimester (*n* = 99).

	1st Trimester	3rd Trimester	*p*
Serum adiponectin (μg/mL) (mean ± SE)	11.6 ± 0. 5	8.7 ± 0.4	0.001

SE: standard error. *p* < 0.05 for significant differences.

**Table 4:** Serum adiponectin levels and its variations from the 1st to the 3rd trimester according to tertiles of adherence to the MedDiet.

	Tertile I (*n* = 51)	Tertile II (*n* = 30)	Tertile III (*n* = 18)
Mean MedScore (1st–3rd trimester)	6.3 ± 0.1	7.7 ± 0.1	9.1 ± 0.2
Adiponectin levels at 1st trimester (μg/mL)	13.1 ± 0.6	11.3 ± 0.9	12.2 ± 0.3
Adiponectin levels at 3rd trimester (μg /mL)	8.6 ± 0.3	8.2 ± 0.6	9.3 ± 1.4
Delta % from the 1st to the 3rd trimester	−34% ± 3%	−15% ± 7%	10% ± 11%
*p*-value vs. tertile I		0.14	0.01

Results are expressed as mean ± error standard; *p* value < 0.05 for significant differences.
